# Use of Slightly Pressurized Carbon Dioxide to Enhance the Antimicrobial Properties of Brines in Naturally Processed Black Table Olives

**DOI:** 10.3390/microorganisms10102049

**Published:** 2022-10-17

**Authors:** Biagi Angelo Zullo, Gino Ciafardini

**Affiliations:** Department of Agricultural, Environmental and Food Sciences, University of Molise, Via de Sanctis, I-86100 Campobasso, Italy

**Keywords:** antimicrobial, brine, carbon dioxide, pressurized CO_2_, table olive

## Abstract

Naturally fermented black table olives are processed at low pH in the presence of high sodium chloride concentrations ranging from 8 to 12% (*w v*^−1^). Reducing the salt content of brine has become an urgent issue as it is responsible for several health and environmental problems. The study aim was to evaluate slightly pressurized CO_2_ (spCO_2_) as a third barrier to microbial growth in naturally processed black table olives with low pH and a reduced NaCl concentration. Based on the assessments performed on a pilot plant scale, an spCO_2_ of 1 bar completely inhibited the growth of the bacteria and molds in the presence of reduced saline concentrations. Furthermore, the amount of yeast decreased in the brine as a function of the NaCl content. Laboratory tests performed under spCO_2_ conditions using a single yeast species from the same habitat confirmed the high sensitivity of some oxidizing yeasts and indicated that the fermenting yeast, *Saccharomyces cerevisiae*, is the most tolerant species. Overall, in the brine of naturally processed olives with a low pH between 4 and 4.2, the antimicrobial properties observed with the high concentrations of NaCl can be achieved with a lower salt dose of 5% (*w v*^−1^) when combined with spCO_2_.

## 1. Introduction

Dissolved carbon dioxide (CO_2_) affects the growth characteristics of several microorganisms, depending on the species, concentration, and gas pressure [1]. Gas overpressure was found to lead to CO_2_ dissolution in water, ultimately resulting in the formation of carbonic acid, according to the following equilibrium reaction [2]:

H_2_O + CO_2_↔ H_2_CO_3_↔ H^+^ + HCO_3_^−^↔ 2H^+^ + CO_3_^2−^

The dissolved CO_2_ lowers the pH of the medium and simultaneously penetrates microbial cells, in which it has specific inhibitory effects [3]. The potential to slow bacterial spoilage through the application of CO_2_ has been increasingly exploited to improve the hygiene of both liquid and solid foods [3,4]. To slow the development of aerobic bacteria and fungi, CO_2_ is currently employed in the food industry for modified atmosphere packing, which can extend the shelf life of commodities, including meat, seafood, cheese, potato chips, and other snacks, under a non-pressurized condition [5]. High-pressure CO_2_ (hpCO_2_), which involves using pressurized CO_2_ between 1 MPa (10 bar) and 50 MPa (500 bar), is applied to devitalize many microorganisms via the non-thermal pasteurization of food [6]. In addition to hpCO_2_, which requires the use of adequate equipment for the application, slightly pressurized CO_2_ (spCO_2_), which involves pressures of less than 1 MPa, can be an alternative strategy for controlling microbial growth. Recent studies revealed the applicability of 0.1 MPa spCO_2_ in managing the aroma profile of wine during alcoholic fermentation [7]. In general, the application of CO_2_ inhibits growth or increases the lag phase and generation time in the growth cycle of microorganisms [8]. These effects vary with the concentration of CO_2_, incubation temperature, microorganisms, and water activity (*aw*) of the medium [9]. However, unlike hpCO_2_, studies pertaining to the use of slightly pressurized CO_2_ to improve the quality of food are lacking. Furthermore, studies are lacking on the use of spCO_2_ to improve the antimicrobial activity of the brines of black table olives processed naturally with reduced concentrations of NaCl. During the production of table olives, the growth of spoilage microorganisms is controlled by reducing the pH and *aw* of the brine using different concentrations of NaCl, depending on the processing system. The methods used to produce table olives include the Sevillan and Californian systems, which involve the chemical treatment of olives with lye, and the natural system, which involves the natural fermentation of the fruits in brine and produces olives as natural black olives [10]. In the natural black table olive processing system, the olives are directly placed in the brine solution containing 8–12% NaCl without any prior debittering pre-treatment. During the fermentation phase, the growth of spoilage microorganisms is inhibited by two barriers, low pH and *aw* due to the high concentration of NaCl [11]. However, reducing the NaCl concentration used in the brine has been strongly recommended. According to the World Health Organization [12], high salt intake via dietary sodium increases the risks of various diseases, such as hypertension and cardiovascular disease [13], and the resulting salt-rich wastewater serves as an environmental pollutant. Thus, reducing the NaCl content in brine during olive fermentation and the final product has garnered attention from the scientific community in the last decade [14]. Furthermore, the reduction in the amount of salt is imperative from both a consumer and an environmental perspective. However, reducing the NaCl level in the brine of black table olives processed naturally causes detrimental effects to the texture, flavor, and shelf life of the final product. Moreover, the inhibition of spoilage microorganisms cannot be guaranteed. Therefore, how the NaCl content of naturally fermented black olives can be reduced without compromising product quality must be determined. In this study, spCO_2_, which is associated with low pH and NaCl content, was introduced for the first time to represent a third barrier to microbial growth. The findings of this study will help to provide a theoretical basis for improving the quality of the low-salt, naturally fermented black table olive. 

## 2. Materials and Methods

### 2.1. Olive Processing on a Pilot Plant Scale 

#### 2.1.1. Short-Term Incubation Tests in Anaerobiosis and spCO_2_ Conditions 

**Figure 1:**
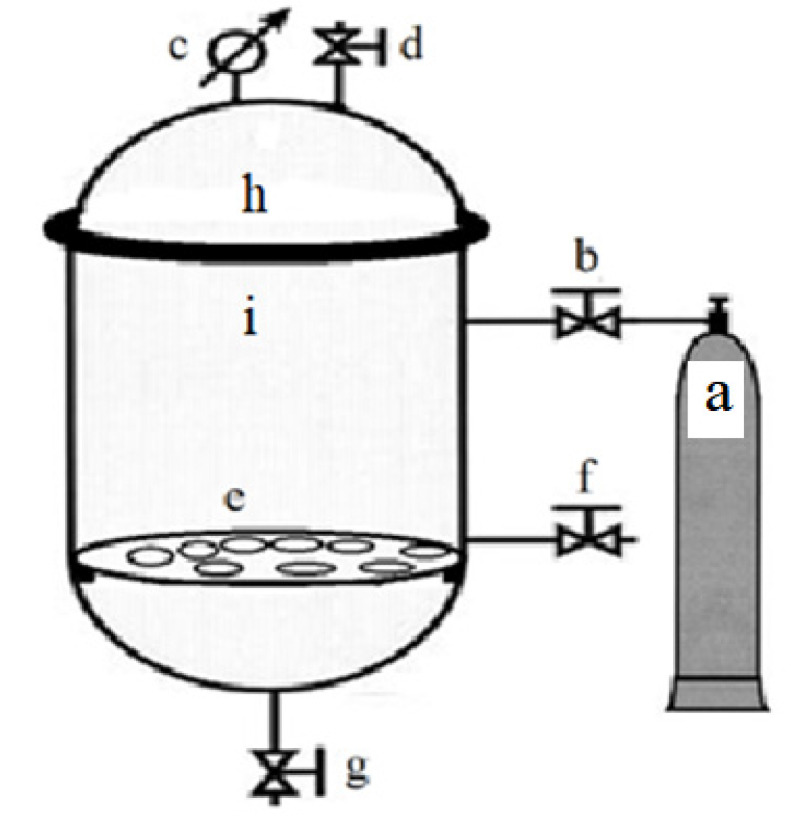
Schematic representation of the pressurized reactor configuration: (a) compressed CO_2_; (b) pressure regulation valve; (c) precision manometer; (d) loading opening for liquids with the decompression valve; (e) removable stand to hold samples; (f) sample intake valve; (g) exhaust valve; and (h) upper cover of the reactor hermetically fixed to the lower part (i) via self-locking.

The experiments were carried out using the fruit of the Leccino cultivar (*Olea europaea* L.). The olives were harvested at the ripening stage, corresponding to a 70% black surface color, from mid-September to mid-October during the 2019–2020 season. Immediately after harvesting, the fruits were transported to the laboratory. After the leaves and other inert materials were removed, the fruits were carefully washed with tap water and placed in 0.5 kg glass vessels. Subsequently, the fruits were covered with brine containing 0, 2, 5, 8, and 11% (*w v*^−1^) NaCl and 0.6% (*w v*^−1^) citric acid. For each type of brine, eight glass vessels were prepared, resulting in a total of 40 samples. Finally, 20 samples were incubated under anaerobic conditions at atmospheric pressure, while the other 20 samples were incubated under spCO_2_ conditions in two 50 L pressurized steel reactors, as shown in Figure 1. 

Anaerobic incubation at atmospheric pressure was achieved by placing the glass vessels without the lid and with the olives in brine on the reactor support (c), following removal of the top cover (h). The lid (h) was then fixed to the lower part (i) via self-locking. Anaerobiosis was established in the reactor by removing the internal air through the CO_2_ gas flow from the pressurized vessel (a) for 5 min following valve activation (b). During the one-month incubation at 18–20 °C, the internal pressure of the reactor was balanced with the external pressure through the decompression valve (d), which discharged the gases derived from the fermentation of the olives in brine. Incubation with spCO_2_ was carried out using a previously described procedure for anaerobic incubation at atmospheric pressure, except that during the one-month incubation, a constant pressure of 1 bar in the reactor was assured. More specifically, compressed CO_2_ (a) was introduced into the reactor containing the samples through the valve (b), and at the beginning of the test, the CO_2_ was allowed to flow out for 5 min through the decompression valve (d) to remove the air present inside the reactor. The valve (d) was then closed, bringing the pressure of the reactor to 1 bar. Finally, the valve (b) was closed. The excess pressure due to the gases from the fermentation of the olives in brine was stabilized at 1 bar through the valve (c). After one month of incubation at 22 °C, the overpressure of 1 bar in the reactor was normalized to zero by activating the exhaust valve (g). The glass vessels with olives in brine were removed from both reactors after lid (h) removal and subjected to microbiological analysis according to the procedure described in Section 2.1.5. Scanning electron microscopy (SEM) was then performed.

#### 2.1.2. SEM Observation 

The microorganisms suspended in the brine fraction were collected via centrifugation at 7000× *g* (Hettich Instruments, Tuttlingen, Germany) for 5 min. The biomass was fixed in 1 mL of 3% (*v v*^−1^) glutaraldehyde (Sigma-Aldrich, St. Louis, MO, USA) in 0.1 M phosphate buffer, pH 7.2, for 12 h. The samples were rinsed thrice in the same buffer and then dehydrated (twice for each solution) using a graded ethanol series (20, 40, 60, 80, 95, and 100%) for 10 min, with a final wash in acetone to ensure a better CO_2_ substitution during the dehydration procedure, at a pressure of 1200 bar. Subsequently, all the samples were dried using CO_2_ critical point drying (Emitech K850), covered with palladium gold (Emitech K550), and observed via SEM using a Zeiss DSM 940 (Zeiss, Rome, Italy).

#### 2.1.3. Long-Term Incubation Tests in Anaerobiosis and spCO_2_Conditions

Long-term incubation tests were performed using olives of the harvested Leccino cultivar. After the removal of foreign material, the olives were washed with tap water, divided into six equal masses of 40 kg each, and distributed in the previously mentioned reactors (Figure 1). Before receiving the olives, the reactors were opened by removing the lid (h) and the removable stand (e) that were used to support the glass vessels in the previous tests. After the olives were placed inside the reactors, the reactors were hermetically sealed by fixing the lid (h) to the basal part with screws and completely filled with brine through the filling tap (d). The brine was formulated based on the results of the microbiological analyses carried out in the 2019–2020 season. The brine used to fill the six reactors contained 5% (*w v*^−1^) NaCl and 0.6% (*w v*^−1^) citric acid, ensuring a ratio of olives/liquid of 4:1.5. Three reactors were used for anaerobic fermentation at atmospheric pressure, while the others were used for fermentation under spCO_2_ conditions. In the first case, the internal pressure of the reactor was balanced with the external pressure through the decompression valve, which, in connection with a special device, removed the gases in the case of overpressure or forfeited the external brine in the case of low pressure. In the reactor with spCO_2_, a constant internal pressure of 1 bar was ensured as in the previous tests. The natural fermentation of Leccino black table olives, carried out under anaerobic and spCO_2_ conditions, lasted 12 months at 9.3–22.5 °C. At the beginning of the experiment and after each month of incubation, the brine samples were collected through the sample-taking valve (f) of each reactor. Brine (400 mL) was withdrawn monthly from each reactor and immediately divided into three sub-samples using sterile glass flasks with stoppers. Each sub-sample was separately subjected to further physico-chemical and microbiological analyses according to the methods described below.

#### 2.1.4. Physico-Chemical Analysis of the Brine

The physico-chemical analysis of the brine samples collected monthly from the reactors involved evaluations of the temperature, pH, titratable acidity, and total polar phenol content. The temperature of the freshly collected brine samples from the reactors was recorded with a digital thermometer equipped with a probe. The pH was evaluated using 50 mL of each subsample and a pH meter with an In Lab Routine Pro probe (Mettler, Toledo, OH, USA). The same brine sample was subsequently used for the analysis of titratable acidity and total polar phenols. Titratable acidity was determined via titration with a 0.1 N NaOH solution. The equivalence point was determined using potentiometry at pH 8.3. The total polar phenol content was evaluated using the Folin–Ciocalteu colorimetric method reported by Ciafardini and Zullo [15]. Each chemical analysis was repeated three times.

#### 2.1.5. Microbiological Analysis of the Brine

Microbiological analysis was performed on the brine samples to enumerate the main microbial groups implicated in natural table olive fermentation, such as total yeasts, total aerobic bacteria (TAB), total anaerobic bacteria (TANB), and total molds [16]. The brine samples were serially diluted by a factor of 10 using sterile Ringer’s solution (0.9% NaCl, *w v*^−1^), and the different dilutions were spread on agar media, as described. Briefly, for the enumeration of total yeasts, serial dilutions of the brine were plated onto MYGP agar medium [17] supplemented with 100 µg mL^−1^ chloramphenicol (Sigma-Aldrich, Milan, Italy) and incubated at 30 °C for 5 days. The TAB content was enumerated after 24 h of incubation at 30 °C on nutrient agar (CM0003, Oxoid, Basingstoke, UK) supplemented with 0.05% (*w v*^−1^) cycloheximide (Sigma-Aldrich, St. Louis, MO, USA) to prevent the growth of yeasts. The TANB content was determined on peptone yeast-extract glucose agar (PYGA) medium comprising the following: phytone 20 g, dextrose 20 g, yeast extract 10 g, NaCl 0.08 g, KH_2_PO_4_ 0.04 g, K_2_HPO_4_ 0.04 g, CaCl_2_ 0.008 g, agar 20 g, and water 1000 mL [18]. The number of bacterial colonies was recorded after 72 h of incubation at 30 °C under anaerobic conditions. The total molds were evaluated after seven days of incubation at 28 °C on glucose yeast extract agar (GYEA; Oxoid, Basingstoke, UK). All plates were visually examined for typical colony types and morphological characteristics, which were recorded with the corresponding growth medium. The results are expressed as Log values of colony-forming units (CFU) per mL of brine (Log CFU mL^−1^). Four replicates were used for each brine sample in the two incubation systems. Colonies of yeasts isolated from brines incubated for one year in anaerobic and spCO_2_ conditions were identified by screening numerous colonies grown on a specific chromogenic medium [19]. The yeast colonies obtained from the microbiological analysis of the brines were used to set up master cultures using CHROMagar Candida medium (BBL, code 4354093, Heidelberg, Germany). After 7 days of incubation at 30 °C, chromogenic characteristics, presence of pseudohyphae, along with cell shape and size were recorded. Based on these characteristics, approximately 1000 yeast colonies were divided into homogenous chromogenic yeast groups. From each homogenous chromogenic yeast group, 10 yeast isolates were randomly selected and analyzed by sequencing approximately 600 base-pair D1/D2 regions of the large (26S) ribosomal subunit, using NL1 and NL4 primers as previously described [20]. The ribosomal sequence obtained from the NL1 primer was compared to those of the yeast species in the public gene database using a BLAST search of the GenBank + EMBL + DDBJ + PDB sequence on the NCBI website http://www.ncbi.nml.nich.gov/blast. The species identified as *Candida adriatica*, *Saccharomyces cerevisiae*, and *Zygotorulaspora mrakii* were subsequently used in the laboratory tests.

### 2.2. Laboratory Tests with Oleuropeinolytic Yeast Species

The aim of the laboratory tests was to determine the effect of spCO_2_ on the growth and oleuropeinolytic activity of the single species of yeasts widely spread in the brines of table olives and olive oil. The yeast species *C. adriatica*, *S. cerevisiae*, and *Z. mrakii* were derived from the tests performed with the pilot plant, while the other species listed in Table 1 were obtained from other sources isolated in previous studies.

#### 2.2.1. Yeast Growth under Different Conditions in the Absence of NaCl 

Yeast cultures grown overnight at 30 °C on MYGPagar medium were used to obtain stocks by suspending a portion of the cells in sterile distilled water. Finally, the O.D._600_ of each culture was measured using a spectrophotometer (Model 6300; Jenway, Essex, UK). The microbial suspensions prepared with the different yeast species had a minimum O.D._600_ shift of 1.478 to a maximum shift of 1.920. The inoculum dose used for each tube varied proportionally from a minimum of 200 µL for the more concentrated stocks to a maximum of 260 µL for less concentrated stocks. Each yeast species was inoculated into 32 test tubes containing MYGP medium, which were closed with a soft cotton cap to promote gaseous exchange with the external environment. After manual light stirring, the tubes were divided into three groups and incubated under aerobic, anaerobic, and spCO_2_ conditions. The aerobic incubation was implemented by placing the cultures directly in the incubator, and the anaerobic conditions were obtained by placing the cultures in GasPak (OxoidAnaeroJar, AG0025). The incubation in spCO_2_ was implemented by placing the cultures on the removable stand (e) of the reactor (Figure 1). After removing the air in the headspace with a flow of CO_2_, an internal pressure of 1 bar was maintained. All the cultures were incubated at 30 °C for 5 days. At the end of the incubation period, the microbial cultures were shaken briefly using a vortex and analyzed using a spectrophotometer at a wavelength of 600 nm. The baseline was set using a fraction of the same yeast culture, which, at the time of inoculation, was stored at −20 °C for the entire duration of the 5-day incubation period.

#### 2.2.2. Yeast Growth under Different Conditions in the Presence of NaCl 

The inhibitory effect of NaCl on yeast growth was evaluated under favorable conditions of aerobiosis and under conditions of stress induced by spCO_2_. The yeasts listed in Table 1 were first grown overnight in MYGPagar and then used to set up the inoculum stocks in a similar manner to the previous test. The amount of inoculum used was equal to that used in the previous test, based on the O.D._600_ of each microbial suspension. The NaCl concentrations tested in the MYGP medium were 0, 5, 8, and 11% (*w v*^−1^). After inoculation in the MYGP medium with different NaCl concentrations, each yeast species was incubated at 30 °C for 5 days under aerobic conditions and in the presence of spCO_2_ in a similar manner to the previous test. Three replicates were used for each salt concentration. At the end of the incubation period, the yeast cultures were analyzed using a spectrophotometer in the same manner as before, and the O.D._600_ was recorded.

#### 2.2.3. Enzymatic Activity of Yeasts Grown under Different Conditions

The oleuropeinolytic capacity of the yeasts was evaluated by determining the activity of β-glucosidase and esterase, the two main enzymes involved in the debittering of naturally processed black table olives. The yeast species assessed were *S. cerevisiae* 2046 and 2074, *Candida boidinii* 1950 and 2076, and *Groenewaldozyma auringiensis* 2054 and 2080. These yeast species were selected based on their widespread diffusion in the brine of table olives [22,23,25,26] and their high oleuropeinolytic activity (Table 1). The yeast strains stored at −40 °C in the presence of 20% (*v v*^−1^) glycerol were transferred to Petri dishes containing MYGPagar medium. After 72 h of incubation at 30 °C, the strains were tested to ascertain their purity and then stored at 4 °C before use in the tests described below.

##### β-Glucosidase and Esterase Activities of Yeasts at pH 4 and 7 in the Absence of NaCl 

The microbial stocks were prepared by placing 0.4 g of biomass of each yeast grown overnight at 30 °C on MYGPagar medium in 10 mL of sterile distilled water. The same microbial stock was used to evaluate the β-glucosidase and esterase activities. The β-glucosidase activity was evaluated at pH 4 and 7 by setting up two stocks of 4-nitrophenyl-β-D-glucopyranoside (4-NPG, Sigma-Aldrich, St. Louis, MO, USA) containing 0.6% (*w v*^−1^) substrate, dissolved in sodium acetate buffer (0.05 M, pH 4) and potassium phosphate buffer (0.05 M, at pH 7), respectively. The enzymatic reaction occurred in a 3 mL solution consisting of 0.5 mL of 4-NPG, 0.2 mL of microbial stock, and 2.3 mL of buffer at pH 4 and 7. A control containing all the ingredients, except 4-NPG, was set up and treated similarly to the other samples. After 3 h of incubation at 30 °C, all the samples were centrifuged at 5000× *g* for 5 min, microfiltered through Millex syringe filters with a pore size of 0.22 µm (Merck Millipore Ltd., Tullagreen, Ireland), and finally analyzed using a spectrophotometer at 410 nm. For the enzymatic reaction occurring in the 0.05 M sodium acetate buffer (pH 4), the pH was increased to 7 with NaOH 1 N before the final reading of the absorbance on the spectrophotometer. The amount of *p*-nitrophenol (*p*-NP) released during the reaction was quantified using a standard curve. The β-glucosidase activity of each sample was measured three times, and the results were expressed as µg p-NP/h/g yeast biomass. The esterase activity was measured at pH 4 and 7 using the same buffers reported; however, the substrate used was *p*-nitrophenyl-acetate (*p*-NPA, Sigma-Aldrich, St. Louis, MO, USA). The enzymatic reaction was allowed by incubating the 3.3 mL mixture, consisting of 0.2 mL of the yeast cell stock reported above, 0.2 mL of 0.15 M *p*-NPA in ethanol, 2.9 mL of 0.05 M acetate buffer pH 4, and 2.9 mL 0.05 M phosphate buffer pH 7, at 30 °C, for 20 min [30]. A *p*-NPA-free control was prepared and processed with the other samples, as previously described, before measuring the absorbance using a spectrophotometer at 410 nm. The esterase activity was expressed in µg *p*-NP/h/g yeast biomass, and each analysis was repeated three times.

##### β-Glucosidase and Esterase Activities of Yeasts Grown in Aerobic and spCO_2_ Conditions in the Presence of NaCl

The oleuropeinolytic activity of the yeasts was analyzed using microbial cultures grown under aerobic conditions and grown in the presence of spCO_2_ to ascertain the influence of CO_2_ on the enzymatic activities of the yeasts. The β-glucosidase and esterase activities were tested at pH 4 in the presence of 0, 5, 8, and 11% (*w v*^−1^) NaCl. Microbial cultures grown in aerobiosis were obtained by incubating yeast inoculated on MYGPagar medium at 30 °C for 5 days. Yeast cultures grown in the presence of spCO_2_ were produced by incubating the yeasts transplanted on the MYGPagar medium at 30 °C for 5 days in the reactor previously mentioned (Figure 1). The procedure was the same as that previously mentioned. At the end of the incubation, the microbial stocks were prepared by suspending 0.4 g of biomass of each yeast in 10 mL of sterile distilled water and were then used for the evaluation of theβ-glucosidase and esterase activities, as reported. The enzymatic reaction of β-glucosidase occurred in a 3 mL solution that comprised 0.5 mL of 4-NPG, 0.2 mL of microbial stock, and 2.3 mL of 0.05 M pH 4 acetate buffer with the mentioned NaCl concentrations. For esterase, the enzyme reaction solution consisted of 2.9 mL of 0.05 M pH 4 acetate buffer with increasing concentrations of NaCl, 0.2 mL of the yeast cell microbial stock, and 0.2 mL of 0.15 M *p*-NPA. The reaction times and spectrophotometer readings at 410 nm were the same as those described above. Three replicates were used for each NaCl concentration.

### 2.3. Statistical Analysis

The Statistical software (version 7.0) was used for data processing (Statsoft for Windows, Tulsa, OK, USA). Comparisons among means were performed with Duncan’s multiple-range tests (one-way ANOVA), and differences were considered significant at *p* < 0.05. 

## 3. Results and Discussion

### 3.1. Pilot Plant Tests

#### 3.1.1. Short-Term Fermentation of Naturally Processed Black Table Olive under Anaerobic and spCO_2_ Conditions

Based on the microbiological analyses, the salt concentration used for the brines of olives in anaerobiosis led to the inhibition of the growth of the microorganisms, particularly bacteria and molds, when employed for the brines of olives processed in the presence of spCO_2_. In the brine of black table olives processed in anaerobiosis, a gradual reduction in the number of yeasts was observed in accordance with the progressive increase in NaCl concentration. Statistically significant differences (*p* < 0.05) were recorded between the concentrations ranging from 5% to 11% (*w v*^−1^) NaCl. Furthermore, the number of TAB decreased as the NaCl content increased, from 0 to 5% (*w v*^−1^), reaching zero at higher concentrations. In contrast, the number of TANB was negative in all the tested samples. Finally, the molds, unlike the yeasts and the TAB, were detected only in the salt-free samples. The microbiological analyses performed using brine samples from olives processed in the presence of spCO_2_, as compared to the previous samples, revealed a lower number of yeasts in the brine samples containing a salt concentration between 0 and 5% (*w v*^−1^) NaCl. However, in the brines richer in salt, the yeasts were absent. Other microorganisms, i.e., TAB, TANB, and molds, were also absent in all the evaluated samples (Table 2). 

**Figure 2:**
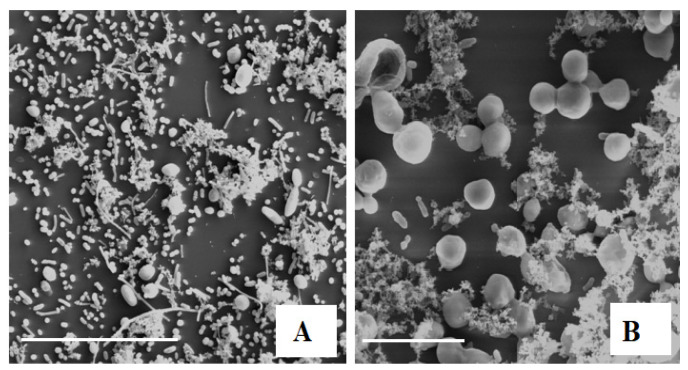
SEM observation of naturally processed black table olive brine with 0% NaCl after one month of incubation in anaerobiosis (**A**) and the presence of slightly pressurized CO_2_ (**B**). Bar 30 µm (**A**) and 10 µm (**B**).

The reduction in microbial growth in the brine of the olives incubated in anaerobiosis for one month can be attributed to the osmotic stress caused by the increasing concentration of NaCl. The inner osmotic pressure of the bacterial cells, termed as “turgor pressure”, is normally maintained at a level higher than that of the medium [31]. To preserve the turgor pressure in a medium rich in solutes, such as brines, and to mitigate the osmotic stress resulting from low water availability, microbial cells tend to increase the concentration of solutes inside them through mechanisms that differ depending on the severity of stress [32]. Under mild osmotic stress, such as the low concentrations of NaCl, the microbial cells are limited to accumulating compatible ionic solutes, which, within certain limits, are tolerated without physiological damage, whereas exposure to severe osmotic stress leads to the synthesis and concentration of specific organic solutes, which are lethal to the cell above a certain threshold [33]. In fact, the number of yeasts revealed in Table 2 was reduced in the brine containing 0 to 11% (*w v*^−1^) NaCl, while bacteria and molds did not grow in the same brine with more than 5% (*w v*^−1^) NaCl, confirming the normal occurrence in the naturally debittered table olives brined with high doses of NaCl [11]. In contrast, the brine from olives fermented in the presence of spCO_2_ displayed significantly higher antimicrobial activity than the previous brine, both in the presence and the absence of NaCl. The higher antimicrobial activity of the brine of fermented olives in the presence of spCO_2_ could be attributed to the greater stress created by the salt and CO_2_ dissolved in the water, which exerts an inhibitory effect on microbial cells at extracellular and intracellular levels. Dissolved CO_2_ is absorbed into the cell membrane and reacts with sterols and unsaturated fatty acids (UFA), thereby changing the order of the membrane by changing its fluidity. The altered properties of the membrane influence its transport characteristics across the membrane [1]. The results in Table 2 show that even in the absence of NaCl, dissolved CO_2_ is active against many microorganisms, including bacteria and molds. At low concentrations of NaCl, yeasts seem to tolerate the stress caused by dissolved CO_2_ in water from an spCO_2_ of 1 bar; however, the addition of NaCl concentrations greater than 5% (*w v*^−1^) creates a level of osmotic stress in the brine when associated with the stress produced by dissolved CO_2_, leading to the complete inhibition of the yeasts. Studies conducted by Dixon and Kell [1] on the effect of pressurized CO_2_ on microbial growth revealed that a CO_2_ partial pressure higher than 6 bar has a negative effect on yeast growth. Scanning electron microscopy analyses performed on the same brine samples with 0% NaCl confirmed the results in Table 2. In fact, the SEM observations indicated a high number of bacteria and yeast in the brines with 0% NaCl, originating from the olives processed in anaerobiosis (Figure 2A). In olives processed in the presence of spCO_2_, a fair presence of yeast was confirmed. Contrary to the results of the microbiological analysis, which revealed the absence of bacteria, a few bacterial cells were present in the olives processed in the presence of spCO_2_ (Figure 2B). 

These results could depend on the modification of the microbial nutrient flow due to the stress created by the spCO_2_, which created a state of starvation in the bacterial cells. Some bacteria have been proposed to enter **a** viable but not culturable state upon starvation [1,34].

#### 3.1.2. Long-Term Processing of Black Table Olives under Anaerobic and spCO_2_ Conditions 

The long-term processing tests were performed for 12 months at room temperature for the olives processed under anaerobic and spCO_2_ conditions using brine with 5% (*w v*^−1^) NaCl and 0.6% (*w v*^−1^) citric acid. The concentrations of the different microorganisms in the brine, which were confirmed on a monthly basis, had different dynamics depending on the incubation system. The brine of the olives processed with anaerobiosis had a greater number of TAB than yeasts and molds when the tests were set up. Later, during incubation, the bacteria gradually reduced until they disappeared after 5 months of storage. The total yeast concentration remained constant in the first 3 months of incubation, and gradually increased until the maximum values were reached after 9 months of storage. However, the prevalence of molds, even at low concentrations, was almost constant throughout the storage period (Table 3). In contrast, the brines of the olives processed in the presence of spCO_2_ showed, on average, a lower number of total yeasts than the previous samples, while the bacteria and molds were not highlighted in any of the analyzed samples. These results suggest that yeasts survive better in the brine of the olives processed in the presence of spCO_2_, including *S. cerevisiae*, which in the last 7 months of incubation showed a prevalence ranging from 80% to 100%. In contrast, the prevalence of *Z. mrakii* and *C. adriatica* was noted in the brine of the olives processed in anaerobic conditions during the first colder months of incubation, and *S. cerevisiae* species only appeared later, with 62–75% prevalence (Table 3). The results of the microbiological analyses in Table 3 confirm those presented in Table 2. Aligning with the first month of anaerobic incubation of the long-term processed olives, the presence of yeasts and bacteria was confirmed in the brine with 5% (*w v*^−1^) NaCl (Table 3), aligning with that found in the brine of the short-term processed olives (Table 2). In the brines of these olives processed in the presence of spCO_2_, no microorganisms were detected in this phase of the incubation characterized by low temperature, including the yeasts that were highlighted after the temperature increase in month 6 of the incubation. However, spCO_2_ inhibited the growth of some undesired oxidizing yeasts, benefiting the oleuropeinolytic and fermenting species known as *S. cerevisiae*, which is involved in the table olive debittering process (Table 3). The different abundances of microorganisms highlighted in the first month of incubation, which are presented in Table 2 and Table 3, substantially depend on the incubation temperatures, which were 22 °C and 10.5 °C in the first and second years of experimentation, respectively.

The results of the physico-chemical analyses of the long-term processed table olive brines did not show variations in terms of fruit defects and the pH and titratable acidity of the brine, whereas significant differences (*p* < 0.05) were recorded with respect to the phenolic compound content. The brine from olives processed in the presence of spCO_2_ had a higher concentration of total phenols than that from the olives processed in anaerobiosis, despite equal debittering of all the corresponding samples of olives at the end of fermentation. During the first three months of incubation, total phenols accumulated in both types of brines. Subsequently, in the brine of the olives processed in the presence of spCO_2_, their concentrations remained almost unchanged. In contrast, in the brine of the olives processed in anaerobiosis, the concentrations were markedly reduced during the hottest months of incubation (Table 4). By comparing the data in Table 4 with those in Table 3, the dynamics of the phenolic compound content can be correlated with the greater or lesser metabolic activity of the microbiota. In fact, owing to the lower number of yeasts found in the brine of the olives processed in the presence of spCO_2_, the phenols failed to be metabolized at the same intensity as those in the brine of the olives processed in anaerobiosis.

### 3.2. Laboratory Tests

Laboratory tests were performed using oleuropeinolytic yeasts that are widely diffused in the brine of naturally debittered black table olives. More specifically, *C. boidinii*, *G. auringiensis*, *Pichia manshurica*, *S. cerevisiae*, and *Wickerhamomyces anomalus* were derived from the brine of the Taggiasca cultivar, whereas *C. adriatica*, *Yamadazyma terventina,* and *Z. mrakii* were derived from the brine and oil of the Leccino cultivar (Table 1). The purpose of the tests was to determine the possible inhibiting effect of spCO_2_ on the growth and the oleuropeinolytic activity of each yeast species normally involved in the naturally processed black table olive.

#### 3.2.1. Yeast Growth under Different Conditions in the Absence of NaCl 

**Figure 3:**
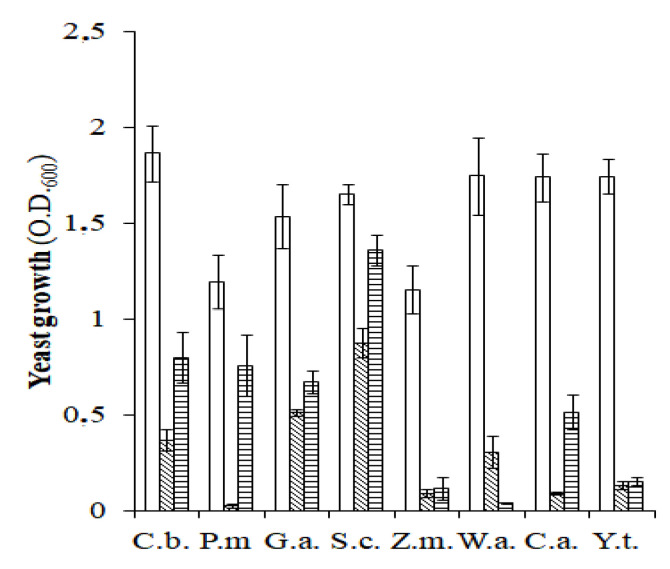
Oleuropeinolytic yeast growth in the absence of NaCl in aerobiosis (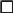), anaerobiosis (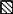), and slightly pressurized CO_2_ (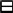) conditions. C.b., *Candida boidinii*; P.m., *Pichia manshurica*; G.a., *Groenewaldozyma auringiensis*; S.c., *Saccharomyces cerevisiae*; Z.m., *Zygotorulaspora mrakii*; W.a., *Wickerhamomyces anomalus*; C.a., *Candida adriatica*; and Y.t., *Yamadazyma terventina*.

The growth tests of yeasts under different conditions, compared to aerobiosis, revealed a strong reduction in the growth of all the species under conditions of anaerobiosis; however, in the presence of spCO_2_, the reduction in growth was more pronounced for some species (Figure 3). 

In aerobiosis, the species with the best growth indices were *G. auringiensis*, *S. cerevisiae*, *W. anomalus*, *C. adriatica*, and *Y. terventina*. Conversely, anaerobiosis significantly and equally reduced the growth of *P. manshurica*, *Z. mrakii*, *C. adriatica*, *Y. terventina*, and *W. anomalus*. The species displaying better tolerance to the anaerobic conditions (in increasing order) were: *C. boidinii*, *G. auringiensis*, and *S. cerevisiae*. Finally, incubation in the presence of spCO_2_, with the exception of *Y. terventina*, *W. anomalus*, and *Z. mrakii*, was less impacted based on yeast growth. In fact, compared to aerobiosis, growth in the presence of spCO_2_ was reduced from 70% to 17%. The yeasts exhibiting better tolerance to spCO_2_ appeared as follows (in increasing order): *C. adriatica*, *G. auringiensis*, *C. boidinii*, *P. manshurica*, and *S. cerevisiae*. The strong incidence of anaerobiosis in the growth of some yeasts is because many of the species shown in Figure 3 are oxidizing yeasts, which in brines are favored by the air-forming superficial films, called flor, which constitutes a spoilage factor as it can worsen the quality of the product [15,35]. Compared with anaerobic conditions, the lower negative incidence of spCO_2_ on the growth of some the species of yeasts shown in Figure 3, including *S. cerevisiae*, may depend on the fact that CO_2_ plays a dual physiological role in microorganisms, as it can stimulate and inhibit cell development. According to some studies, in *S. cerevisiae* grown under anaerobic conditions the presence of abundant exogenous CO_2_ provided 6.5% of the yeast total carbon when grown on glucose, whereas under aerobic conditions, 2.6% of the yeast carbon was derived from exogenous CO_2_ [36,37].

#### 3.2.2. Yeast Growth under Different Conditions in the Presence of NaCl

**Figure 4:**
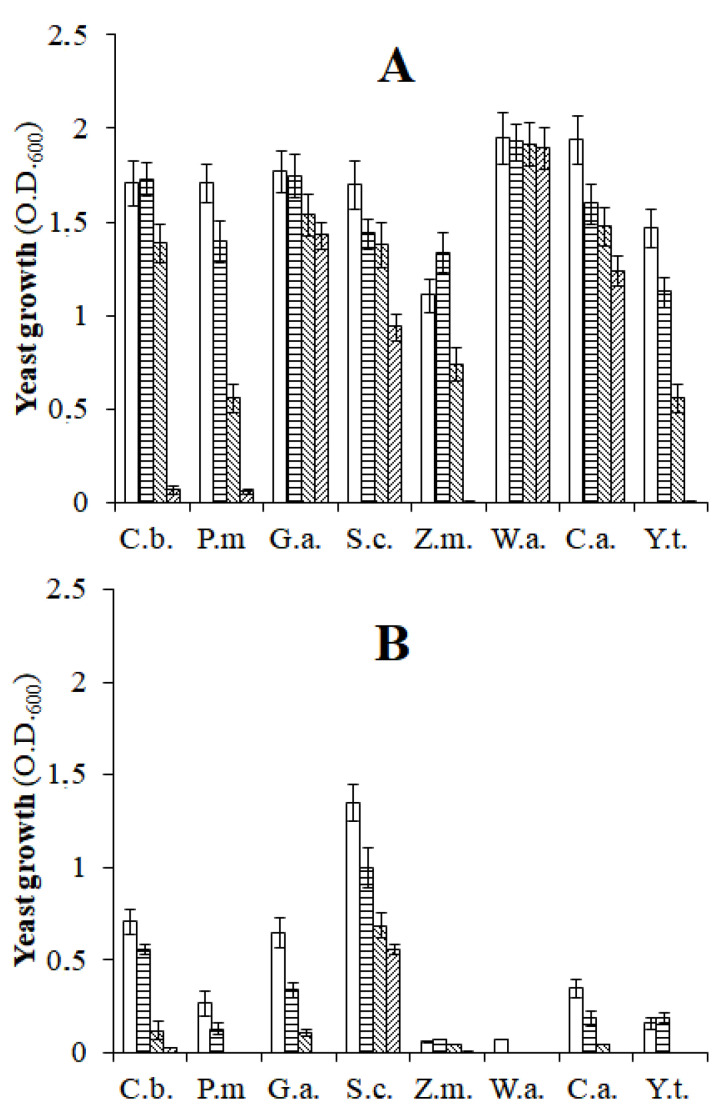
Oleuropeinolytic yeast growth in aerobiosis (**A**) and slightly pressurized CO_2_ (**B**) conditions in the presence of 0 (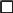), 5 (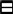), 8 (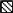), and 11% (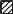) of NaCl. C.b., *Candida boidinii*; P.m., *Pichia manshurica*; G.a., *Groenewaldozyma auringiensis*; S.c., *Saccharomyces cerevisiae*; Z.m., *Zygotorulaspora mrakii*; W.a., *Wickerhamomyces anomalus*; C.a., *Candida adriatica*; Y.t., *Yamadazyma terventina*.

The tests investigating the growth of yeasts in the presence of NaCl and incubated under favorable conditions of aerobiosis and in the presence of spCO_2_ revealed different results depending on the species of yeast examined. Under aerobic conditions, *W. anomalus* tolerated all the concentrations of NaCl in the medium, while other species, namely *C. boidinii*, *G. auringiensis*, and *Z. mrakii,* tolerated the minimum NaCl concentration of 5% (*w v*^−1^) but were heavily inhibited by higher NaCl concentrations. The growth of the remaining species was inhibited almost proportionally to the NaCl concentration used (Figure 4A). According to other findings, the data in Figure 4A reveal that most oleuropeinolytic yeast species are inhibited by NaCl and that an increase in concentration causes a reduction in growth, an elongation of the growth curve, and a lowering of the absorption of sugars [38]. The cell wall of yeast is semipermeable; when a significant amount of NaCl is present in the vicinity, the yeast cell wall releases water by activating the defense mechanisms described earlier in this study. As this water is necessary for cellular activities, its release will slow down the growth of yeast. The growth of the same yeast species incubated in the presence of spCO_2_ was significantly lower than that in the previous experiment (Figure 4A), both in the absence of NaCl and the presence of increasing doses of salt. The reduction in the growth of yeasts in the salt-free substrate can be solely attributed to the inhibitory activity of spCO_2_, which strongly impacted *Z. mrakii*, *W. anomalus*, and *Y. terventina*. The following species displayed better tolerance (in increasing order): *P. manshurica*, *C. adriatica*, *C. boidinii*, *G. auringiensis*, and *S. cerevisiae.* The addition of salt to the substrate further reduced the growth of yeast incubated in the presence of spCO_2_. In fact, the minimum NaCl concentration of 5% (*w v*^−1^) completely inhibited the growth of *Z. mrakii* and *W. anomalus*. In contrast, the following species exhibited better tolerance (in increasing order): *P. manshurica*, *C adriatica*, *G. auringiensis*, *C. boidinii*, and *S. cerevisiae* (Figure 4B). Higher NaCl concentration (11%) markedly reduced the growth of all species, with the exception of *S. cerevisiae,* which was the most tolerant species (Figure 4B). Figure 4A,B confirms the data in Table 2, as a lower abundance of yeast was observed in the brines with 5% (*w v*^−1^) NaCl, when the Leccino black table olives were processed for one year in the presence of spCO_2_. Similarly, the better salt tolerance shown by *S. cerevisiae* in the laboratory when incubated under spCO_2_ conditions (Figure 4B) justifies the prevalence of this species in the brine of the olives processed in the presence of spCO_2_ (Table 3).

#### 3.2.3. Enzymatic Activity of Yeast Grown under Different Conditions

**Figure 5:**
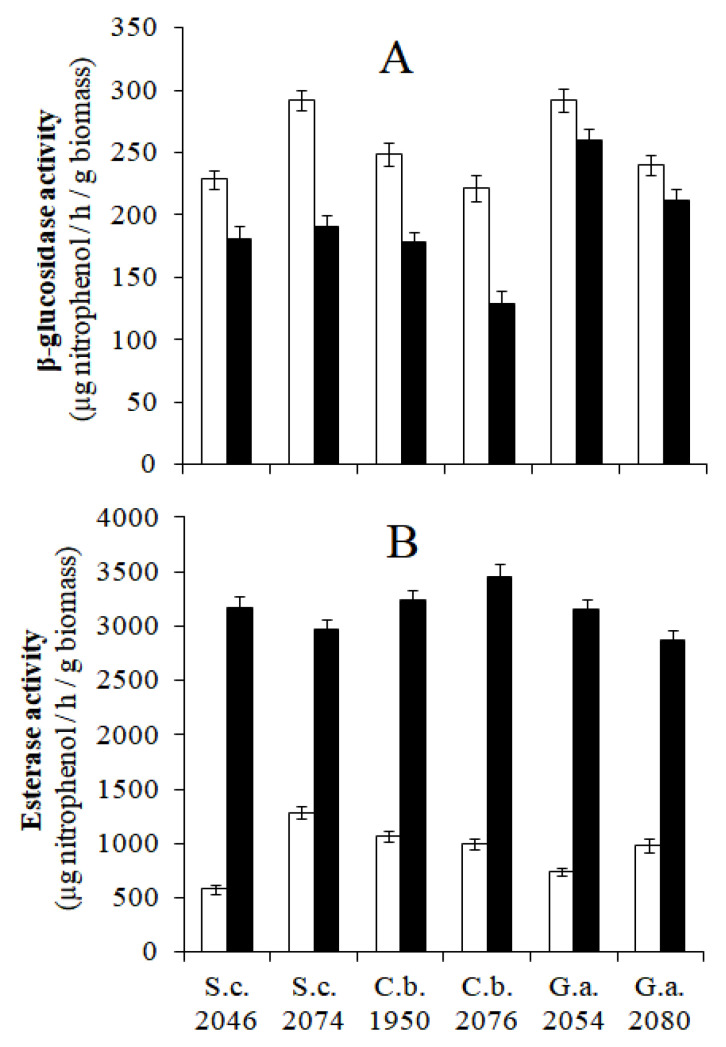
β-glucosidase (**A**) and esterase activities (**B**), at pH 4 (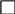) and 7 (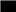) of six yeast strains from naturally processed black table olives.

The oleuropeinolytic activity of yeast cells at pH 4 and 7 in the absence of NaCl involved the enzymatic analysis of β-glucosidase and esterase. Aligning with other studies, the β-glucosidase activity was higher at pH 4 in all the analyzed yeast strains [39]. The differences in pH mainly influenced strains 2046 and 2074 of *S. cerevisiae* and strains 1950 and 2076 of *C. boidinii*. In contrast, the strains belonging to the *G. auringiensis* species did not undergo significant changes (Figure 5A). The esterase activity displayed an opposite behavioral trend to that of the β-glucosidase enzyme as the enzymatic activity recorded at pH 4 was significantly lower than that at pH 7. However, at both pH values, no significant differences related to the yeast strain were observed (Figure 5B).

**Figure 6:**
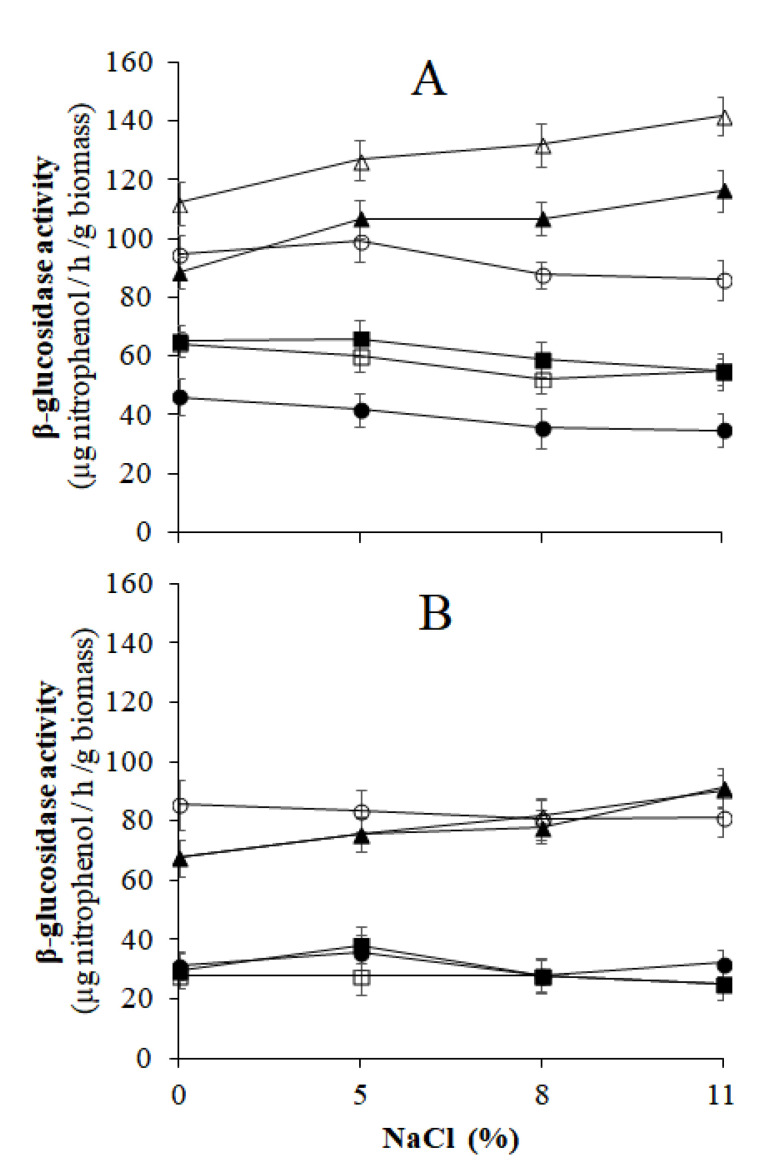
β-glucosidase activity of oleuropeinolytic yeast strains grown under aerobic (**A**) and slightly pressurized CO_2_ (**B**) conditions, evaluated in the presence of NaCl. (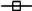, *S. cerevisiae* 2046; 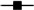, *S. cerevisiae* 2074; 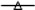, *C. boidinii* 1950; 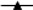, *C. boidinii* 2076; 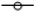, *G. auringiensis* 2054; 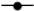, *G. auringiensis* 2080).

The data described indicate that the acidic environment (pH 4–4.2) of the brine of naturally processed black table olives, obtained by external acidification or the activity of lactic acid bacteria, plays a double role in favoring the hydrolysis of oleuropein promoted by β-glucosidase and in meeting the health and hygiene requirements recommended by the International Olive Council (IOC) regulations [40]. The activity of β-glucosidase was evaluated at pH 4; however, in the presence of salt, there were small variations related to the increase in NaCl concentration in all the yeast strains studied (Figure 6). Compared with the results observed during previous aerobiosis growth, cell incubation in the presence of spCO_2_ decreased the synthesis of β-glucosidase by approximately 50% in all the strains of yeast tested (Figure 6A,B). 

**Figure 7:**
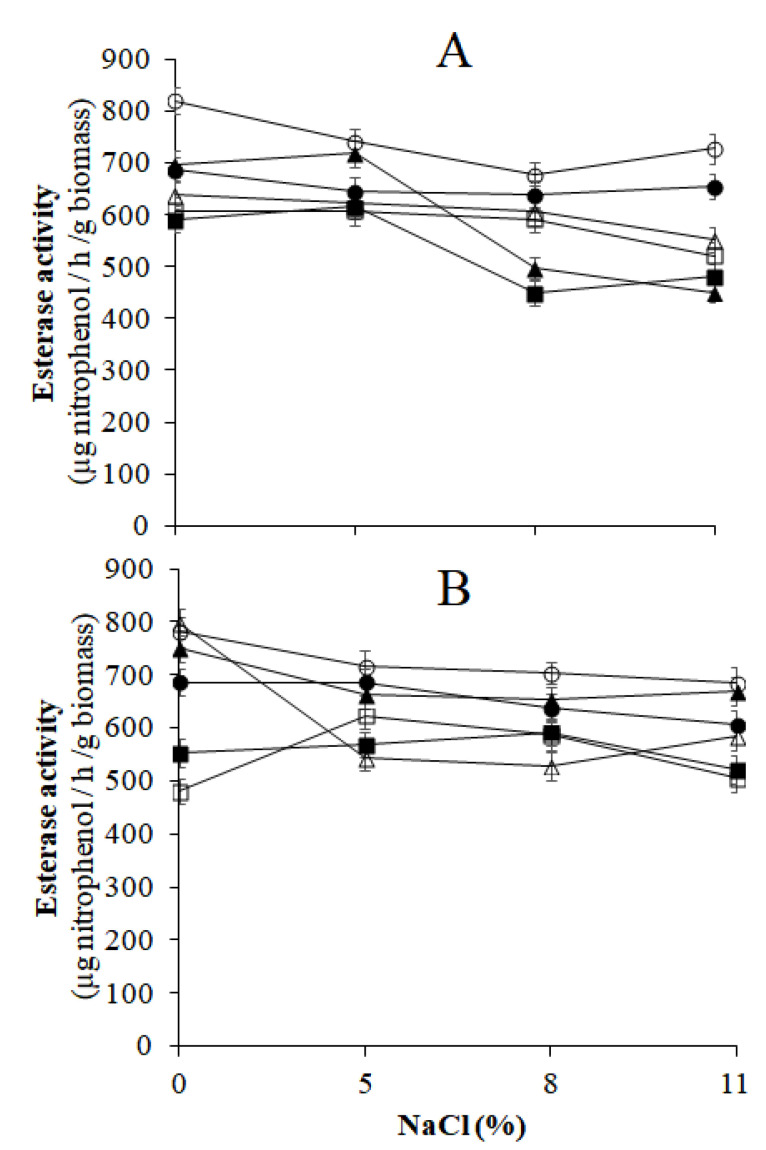
Esterase activity of oleuropeinolytic yeast strains grown in aerobiosis (**A**) and slightly pressurized CO_2_ (**B**) conditions, evaluated in the presence of NaCl. (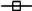, *S. cerevisiae* 2046; 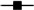, *S. cerevisiae* 2074; 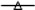, *C. boidinii* 1950; 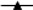, *C. boidinii* 2076; 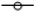, *G. auringiensis* 2054; 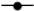, *G. auringiensis* 2080).

These results align with those of other studies, which revealed that the synthesis of β-glucosidase in some yeasts, such as *S. cerevisiae,* was enhanced by the aerobic conditions of growth [41]. In contrast, the esterase activity was not negatively affected by spCO_2_, even if some yeast strains did not tolerate high doses of NaCl in the reaction environment (Figure 7A,B). 

The results shown in Figure 6 and Figure 7 indicate that the oleuropeinolytic activity of the yeasts tested in this study was not completely inhibited in the NaCl-rich brine of naturally processed black table olives incubated in the presence of spCO_2_; thus, the technological role of yeasts in the naturally processed table olives remains unchanged.

## 4. Conclusions

The outcome of the fermentation tests performed with the pilot plant and those carried out in the laboratory with the oleuropeinolytic yeasts isolated from the same habitat revealed that spCO_2_ could enhance the antimicrobial activity of the brine used in naturally processed black table olives. In accordance with the “Hurdles Concept”, the addition of spCO_2_ as a third barrier to microbial growth increased the antimicrobial activity at pH 4 and that of the reduced NaCl concentration of 5% (*w v*^−1^) to a greater extent than that at pH 4 in combination with a high salt concentration of 12% (*w v*^−1^), which is normally used in the brine of naturally processed black table olives. These promising results on the control of the bacteria, molds, and oxidizing yeasts responsible for olive spoilage emphasize, for the first time, the possible use of spCO_2_ in naturally processed black table olives in the presence of a reduced NaCl content. The use of spCO_2_ might represent a new and promising approach for improving product quality by reducing the concentration of NaCl in brine and fruits, increasing the phenol content in the processed fruits, reducing the defects linked to spoilage microorganisms, preventing microbial films on the surface of the brines, and reducing the pollution caused by salt-rich wastewater. However, other studies are required to investigate and confirm these aspects.

## Figures and Tables

**Table 1:** Oleuropeinolytic yeasts from table olive brine and virgin olive oil used in the laboratory tests.

Yeast	Source	Oleuropeinolytic Properties	Reference
*Candida adriatica*	Leccino virgin olive oil	+	[21,22], this study
	Manzanilla, Leccino table olive brine		
*Candida boidinii*	Bosana, Manzanilla, Taggiasca table olive brine	++	[22,23], unpublished data
*Groenewaldozyma auringiensis*	Taggiasca table olive brine	+	[Unpublished data]
*Pichia manshurica*	Kalamata, Taggiasca table olive brine	++	[24,25]
*Saccharomyces cerevisiae*	Bosana, Nocellara messinese, Taggiasca, Leccino table olive brine	+	[23,25,26], this study
*Wickerhamomyces* *anomalus*	Gemlik, Bosana, Nocellara messinese, Taggiasca table olive brine	+	[23,26,27], unpublished data
*Yamadazyma terventina*	Leccino, Frantoio, and Moraiolo virgin olive oil	+	[28,29]
*Zygotorulaspora mrakii*	Bosana, Manzanilla, Taggiasca, Leccino table olive brine	+	[22,23,25], this study

+, moderate activity; ++, high activity.

**Table 2:** Microbial growth in the brine of naturally processed Leccino table olives with different NaCl concentrations after one month of incubation in anaerobiosis and slightly pressurized CO_2_ conditions.

NaCl Concentration (%, *w v*^−1^)	Anaerobiosis			spCO_2_^2^		
	Total Yeasts ^1^	Total Bacteria	Total Moulds	Total Yeasts	Total Bacteria	Total Moulds
0	5.77±0.18 ^a^	6.34 ± 0.25 ^a^	1.85 ± 0.19	3.74 ± 0.07 ^a^	n.d.	n.d.
2	5.96 ± 0.01 ^a^	5.94 ± 0.12 ^ab^	n.d.	2.65 ± 0.09 ^b^	n.d.	n.d.
5	4.88 ± 0.13 ^ab^	4.37 ± 0.14 ^b^	n.d.	1.65 ± 0.03 ^c^	n.d.	n.d.
8	4.19 ± 0.07 ^b^	n.d.	n.d.	n.d.	n.d.	n.d.
11	3.62 ± 0.08 ^c^	n.d.	n.d.	n.d.	n.d.	n.d.

^1^ Log CFU mL^−1^; ^2^ spCO_2_, slightly pressurized CO_2_; mean ± standard deviation (*n* = 4); n.d., not detected (value < detection limit of the method). Values in columns with different letters are significantly different from each other at *p* < 0.05.

**Table 3:** Microbial growth in brines of naturally processed Leccino table olives with 5% (*w v*^−1^) NaCl and 0.6% (*w v*^−1^) citric acid during one year of incubation at room temperature in anaerobiosis and slightly pressurized CO_2_ conditions.

Month	Brine Temperature (°C)	Total Yeasts (Log CFU/mL)		Preeminent Yeast Species ^2^		Total Aerobic Bacteria (Log CFU/mL)		Total Molds (Log CFU/mL)	
		Anaerobiosis	spCO_2_^1^	Anaerobiosis	spCO_2_	Anaerobiosis	spCO_2_	Anaerobiosis	spCO_2_
0		0.89 ± 0.12 ^d^	0.89 ± 0.12 ^d^			2.49 ± 0.02 ^a^	2.49 ± 0.02	1.09 ± 0.20 ^b^	1.09 ± 0.20
1	10.5	0.58 ± 0.06 ^d^	n.d.		n.d.	1.05 ± 0.03 ^b^	n.d.	n.d.	n.d.
2	9.3	0.70 ± 0.02 ^d^	n.d.	*Z.m.* (60)	n.d.	1.25 ± 0.08 ^b^	n.d.	n.d.	n.d.
3	9.7	0.80 ± 0.02 ^d^	n.d.	*C.a.* (50)	n.d.	0.75 ± 0.01 ^c^	n.d.	n.d.	n.d.
4	12.2	1.60 ± 0.10 ^c^	n.d.	*C.a.* (52)	n.d.	0.62 ± 0.05 ^c^	n.d.	1.47 ± 0.29 ^ab^	n.d.
5	14.5	1.88 ± 0.23 ^c^	n.d.	*Z.m.* (51)	n.d.	n.d.	n.d.	1.90 ± 0.19 ^a^	n.d.
6	15.3	3.60 ± 0.32 ^b^	2.30 ± 0.22 ^b^	*C.a.* (58)	*S.c.* (80)	n.d.	n.d.	2.03 ± 0.23 ^a^	n.d.
7	18.5	4.33 ± 0.15 ^ab^	3.48 ± 0.11 ^a^	*C.a.* (54)	*S.c.* (85)	n.d.	n.d.	1.87 ± 0.10 ^a^	n.d.
8	20.8	4.71 ± 0.22 ^ab^	3.05 ± 0.02 ^a^	*C.a.* (57)	*S.c.* (87)	n.d.	n.d.	1.97 ± 0.12 ^a^	n.d.
9	22.5	5.31 ± 0.22 ^a^	3.32 ± 0.12 ^a^	*S.c.* (60)	*S.c.* (100)	n.d.	n.d.	1.77 ± 0.09 ^a^	n.d.
10	16.5	5.60 ± 0.12 ^a^	3.41 ± 0.10 ^a^	*S.c.* (62)	*S.c.* (100)	n.d.	n.d.	1.80 ± 0.20 ^a^	n.d.
11	12.5	5.80 ± 0.09 ^a^	3.65 ± 0.07 ^a^	*S.c.* (70)	*S.c.* (100)	n.d.	n.d.	1.74 ± 0.11 ^a^	n.d.
12	11.8	5.98 ± 0.05 ^a^	3.76 ± 0.11 ^a^	*S.c.* (75)	*S.c.* (100)	n.d.	n.d.	1.84 ± 0.11 ^a^	n.d.

^1^ spCO_2_, slightly pressurized CO_2_; ^2^ % compared to the total yeast; mean ± standard deviation (*n* = 9); n.d., not detected (value < detection limit of the method); *Z.m.*, *Zygotorulaspora mrakii*; *C.a.*, *Candida adriatica*; *S.c.*, *Saccharomyces cerevisiae*. Values in columns with different letters are significantly different from one another at *p* < 0.05.

**Table 4:** Physico-chemical characteristics of naturally processed Leccino table olive brine with 5% NaCl and 0.6% citric acid during one year of incubation under anaerobic and slightly pressurized CO_2_ conditions.

Month	pH		Titratable Acidity (g Lactic Acid/L)		Total Phenols (mg CAE ^1^/mL)		
	Anaerobiosis	spCO_2_ ^2^	Anaerobiosis	spCO_2_	Anaerobiosis	spCO_2_	∆ ^3^
1	4.10 ± 0.10	3.44 ± 0.09	7.16 ± 0.12	7.47 ± 0.11	2.46 ± 0.07 a ^4 ^ a ^5^	1.08 ± 0.09 b b	−56
2	4.09 ± 0.08	3.83 ± 0.13	7.29 ± 0.12	7.48 ± 0.13	2.49 ± 0.01 a a	2.21 ± 0.01 ab a	−11
3	3.98 ± 0.08	3.90 ± 0.10	7.40 ± 0.15	7.35 ± 0.12	2.50 ± 0.03 a a	2.45 ± 0.04 a a	−2
4	4.08 ± 0.08	3.93 ± 0.10	7.35 ± 0.12	7.25 ± 0.11	1.90 ± 0.02 a b	2.50 ± 0.02 a a	32
5	4.00 ± 0.08	3.95 ± 0.11	7.43 ± 0.17	7.45 ± 0.15	1.98 ± 0.04 a b	2.65 ± 0.03 a a	34
6	3.88 ± 0.08	3.98 ± 0.10	7.40 ± 0.13	7.55 ± 0.12	2.00 ± 0.01 a b	2.80 ± 0.01 a a	40
7	3.96 ± 0.12	4.00 ± 0.11	7.77 ± 0.19	7.73 ± 0.20	2.30 ± 0.03 a b	2.90 ± 0.01 a a	26
8	3.90 ± 0.04	4.02 ± 0.07	7.70 ± 0.17	7.23 ± 0.14	2.23 ± 0.04 a b	3.08 ± 0.03 a a	38
9	3.88 ± 0.08	3.93 ± 0.10	7.43 ± 0.12	7.34 ± 0.10	2.20 ± 0.02 a b	2.75 ± 0.02 a a	25
10	3.98 ± 0.08	3.93 ± 0.10	7.30 ± 0.17	7.05 ± 0.15	1.90 ± 0.025 ab b	2.65 ± 0.03 a a	39
11	4.20 ± 0.06	4.00 ± 0.11	6.98 ± 0.10	7.29 ± 0.14	1.85 ± 0.02 b c	2.53 ± 0.01 a a	37
12	4.23 ± 0.10	4.16 ± 0.13	7.40 ± 0.14	8.10 ± 0.12	1.72 ± 0.01 b c	2.50 ± 0.04 a a	45
Mean value	4.02 ± 0.12	3.92 ± 0.17	7.38 ± 0.21	7.44 ± 0.27	2.13 ± 0.27	2.51 ± 0.50	

^1^ CAE, caffeic acid equivalent; ^2^ spCO_2_, slightly pressurized CO_2_;^3^ percentage change of total phenols found in the brines from the spCO_2_ fermentation condition compared to that in the anaerobic condition; ^4^ values in columns with different letters are significantly different from one another at *p <* 0.05; ^5^ values in lines with different letters are significantly different from each other at *p* < 0.05.

## Data Availability

Not applicable.
